# Supplementary material for: Comprehensive hallmark gene sequence, genomic and structural analysis clarifies new and established taxa within the Picornavirales
Source: Virus Evol. 2026 Apr 20;12(1):veag023. doi: 10.1093/ve/veag023 (PMC13155101; doi:10.1093/ve/veag023)
Supplement: Supplementary_materials_veag023 [file supplementary_materials_veag023.zip › SI6_families_table/SI_6.docx]

Comprehensive hallmark gene sequence, genomic and structural analysis clarifies new and established taxa within the *Picornavirales*

Richard Mayne, Donald B. Smith, Katherine Brown, *et al*. (2026) *Virus Evolution*

Supplementary Information 6

| **Family** | **Host range** | **Genome** |
| --- | --- | --- |
| *Caliciviridae* | Mammalia | 7–8 kb, monopartite polycistronic |
| *Dicistroviridae* | Arthropoda | 9–10 Kb, monopartite bicistronic |
| *Iflaviridae* | Arthropoda | 9–11 kb, monopartite monocistronic |
| *Marnaviridae* | Var. marine protists | 9–10 kb, monopartite monocistronic |
| *Picornaviridae* | Animalia | 7–10 kb, monopartite monocistronic |
| *Polycipiviridae* | Arthropoda | 10–12 kb, monopartite polycistronic |
| *Secoviridae* | Plantae | 9–14 kb, mono-/bipartite monocistronic |
| *Solinviviridae* | Arthropoda | 10–11 kb, monopartite polycistronic |
| *Noraviridae* | *Drosophila* | 12 kb, monopartite polycistronic |
| NF-1 | Freshwater macrophytes, yellow catfish (M) | 7-11 kb, monopartite monocistronic |
| NF-2 | Animalia | 10 kb, monopartite monocistronic |
| NF-3 | Plantae, Arthropoda*,* bat and parrot faeces (M) | 6-9 kb, monopartite bi-/polycistronic |
| NF-4 | Arthropoda | 10 kb, monopartite monocistronic |
| NF-5 | Bat, bird, rat and hedgehog faeces (M) | 8-11 kb, monopartite monocistronic |
| NF-6 | Sewage, platyhelminthes, molluscs, wasps, bat faeces (M) | 6-11 kb, monopartite polycistronic |

Table S6.1. Expansion of Table 1 in main article, to show range and genome organisation for newly proposed *Picornavirales* families. Host ranges for new families listed as sequence source, rather than confirmed infection. (M) = metagenomically derived
