# Supplementary material for: Comprehensive hallmark gene sequence, genomic and structural analysis clarifies new and established taxa within the Picornavirales
Source: Virus Evol. 2026 Apr 20;12(1):veag023. doi: 10.1093/ve/veag023 (PMC13155101; doi:10.1093/ve/veag023)
Supplement: Supplementary_materials_veag023 [file supplementary_materials_veag023.zip › Supplementary information.docx]

# Supplementary information

1. GRAViTy-V2 run parameters file and complete input data VMR.
2. Tabulated classifications.
3. Tabulated classification summary.
4. All rectangular trees in PDF format.
5. GRAViTy-V2 full output tables.
6. Expanded table 1 to show host range and genomic features of new families.
