# Supplementary figures and images for: Comprehensive hallmark gene sequence, genomic and structural analysis clarifies new and established taxa within the Picornavirales
Source: Virus Evol. 2026 Apr 20;12(1):veag023. doi: 10.1093/ve/veag023 (PMC13155101; doi:10.1093/ve/veag023)

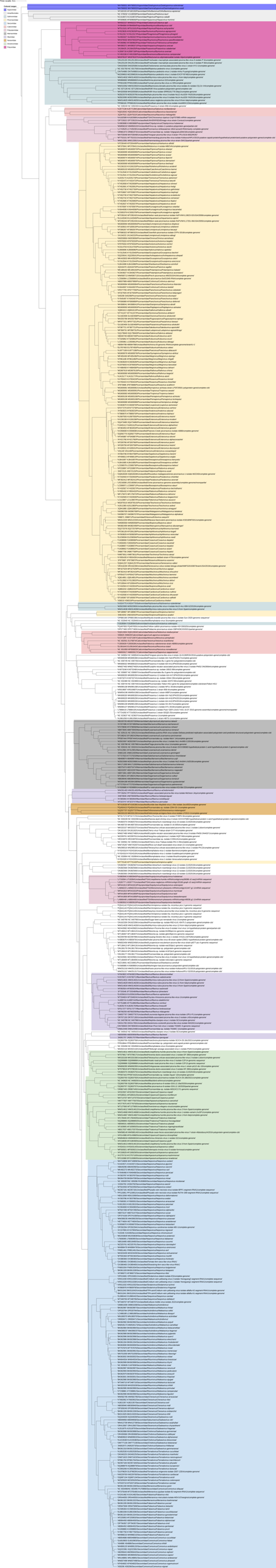

Supplement: Supplementary_materials_veag023 [file supplementary_materials_veag023.zip › SI4_rectangular_trees/SI4_gravity_rectangle.pdf]

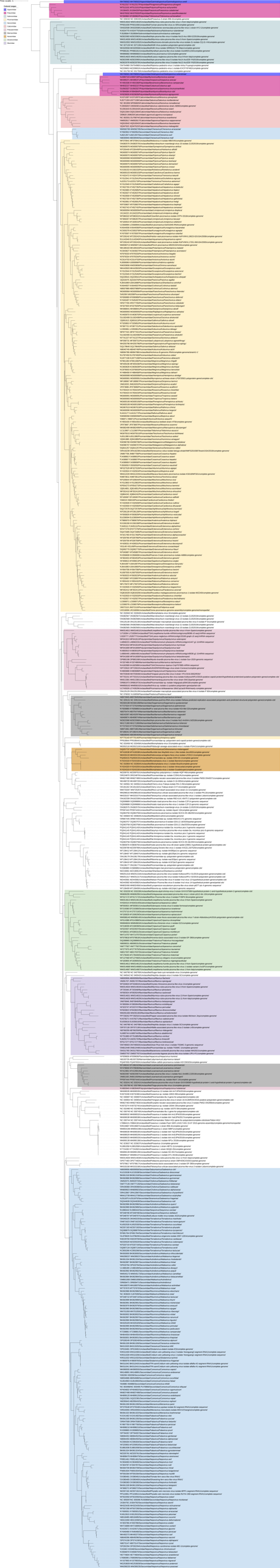

Supplement: Supplementary_materials_veag023 [file supplementary_materials_veag023.zip › SI4_rectangular_trees/SI4_helicase_rectangular.pdf]

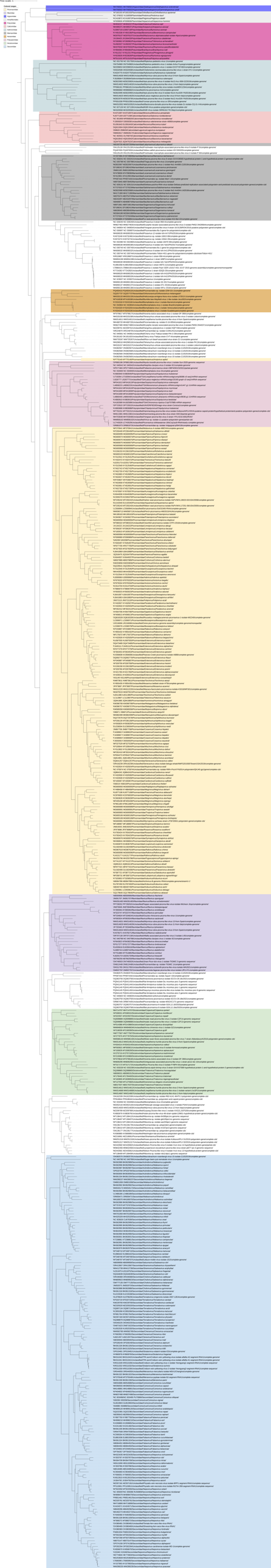

Supplement: Supplementary_materials_veag023 [file supplementary_materials_veag023.zip › SI4_rectangular_trees/SI4_rdrp_rectangular.pdf]

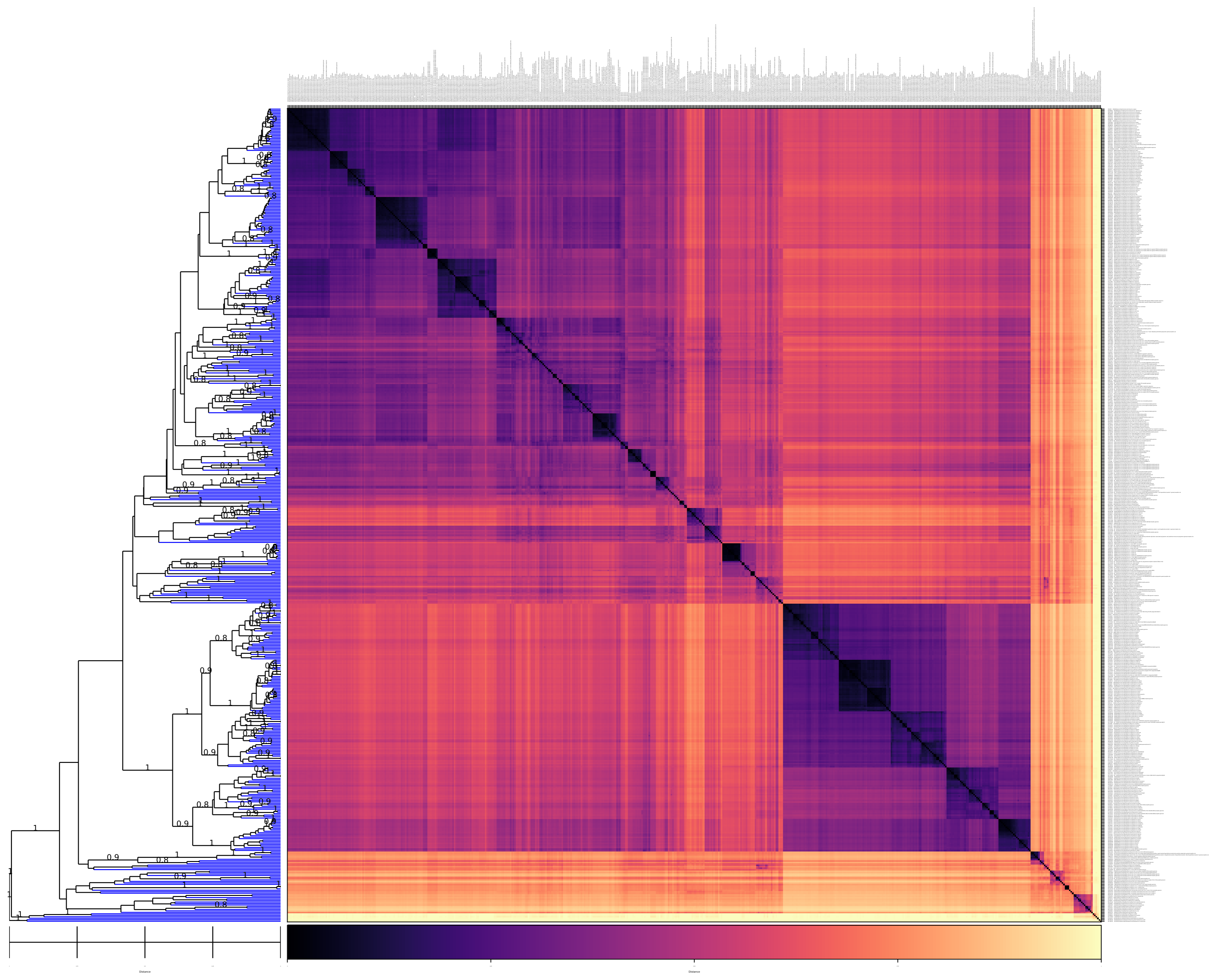

Supplement: Supplementary_materials_veag023 [file supplementary_materials_veag023.zip › SI5_gravity_output/SI5_GRAViTy_heatmap.pdf]

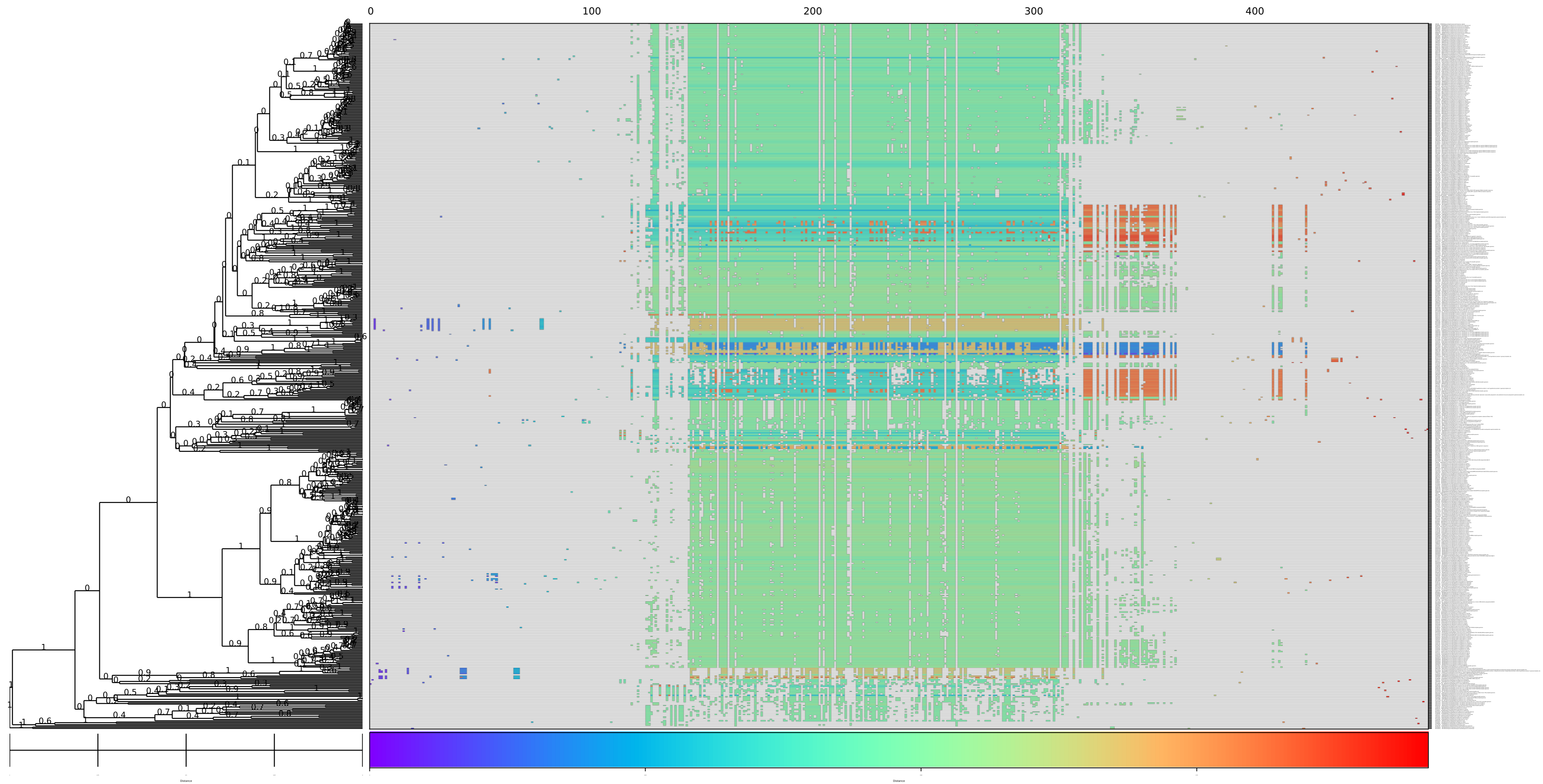

Supplement: Supplementary_materials_veag023 [file supplementary_materials_veag023.zip › SI5_gravity_output/SI5_Pphmm_locations.pdf]

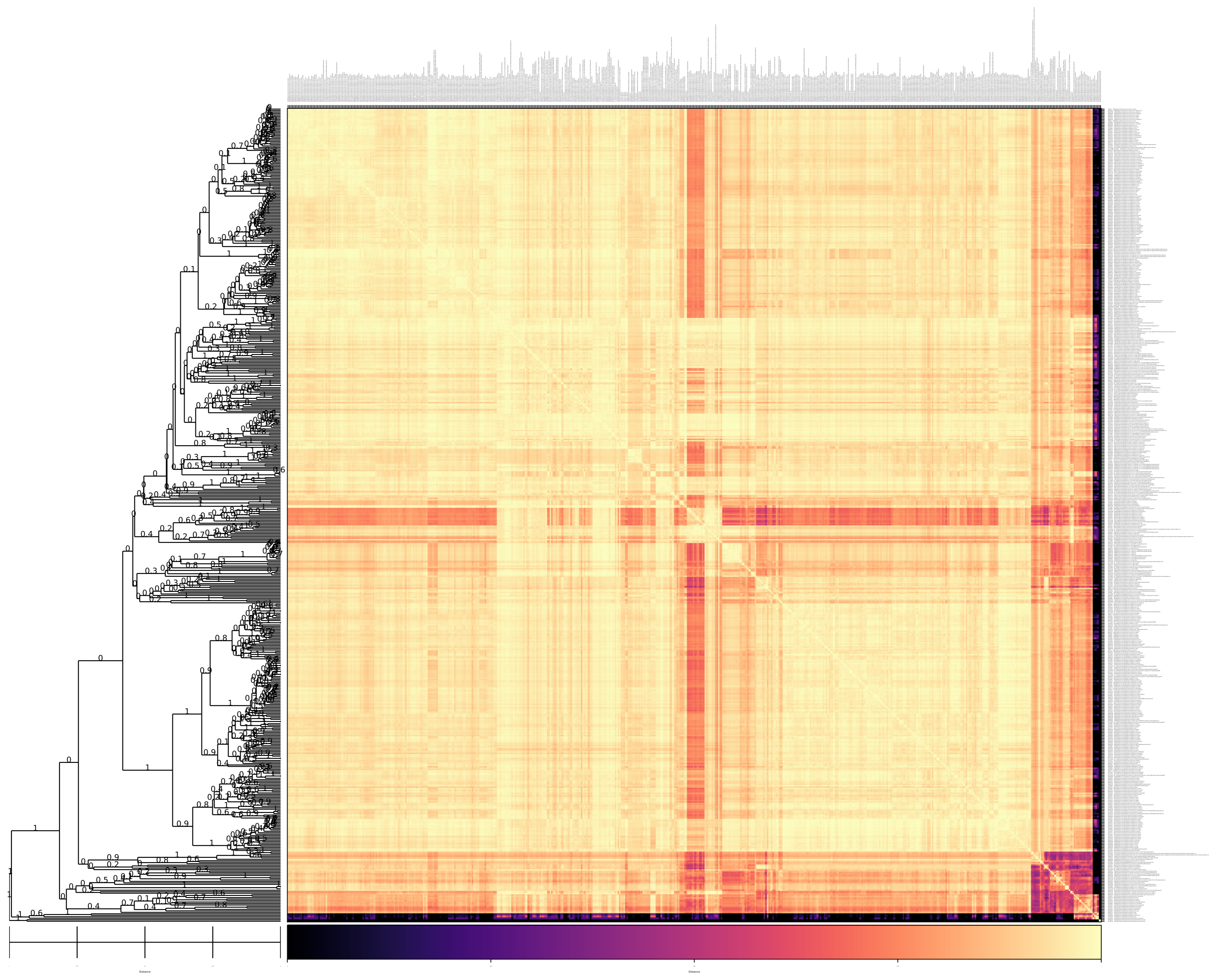

Supplement: Supplementary_materials_veag023 [file supplementary_materials_veag023.zip › SI5_gravity_output/SI5_Shared_norm_pphmm_ratio_matrix.pdf]
