# Supplementary material for: Comprehensive hallmark gene sequence, genomic and structural analysis clarifies new and established taxa within the Picornavirales
Source: Virus Evol. 2026 Apr 20;12(1):veag023. doi: 10.1093/ve/veag023 (PMC13155101; doi:10.1093/ve/veag023)
Supplement: Supplementary_materials_veag023 [file SI4_colabfold_rectangular.pdf › SI4_rectangular_trees/SI4_colabfold_rectangular.pdf]

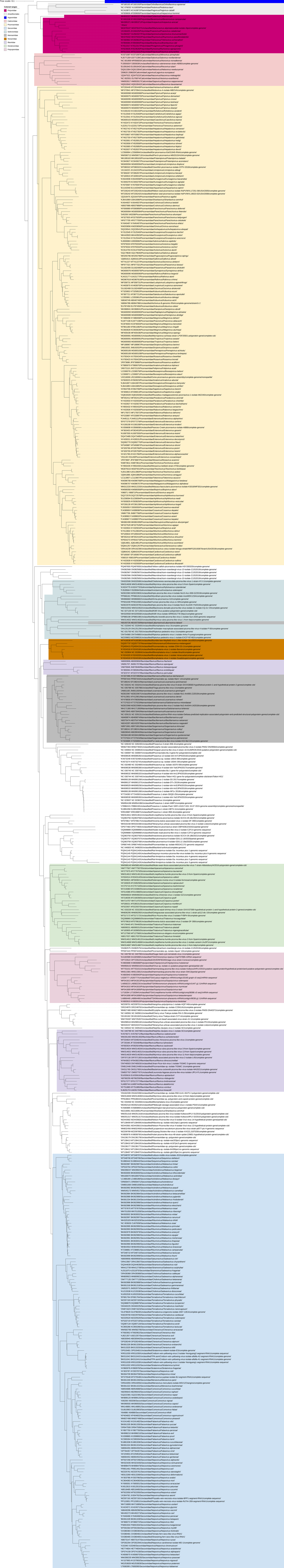

Tree scale: 0.1
Colored ranges
Potyviridae
Amarillovirales
Hypoviridae
Caliciviridae
Picornaviridae
Secoviridae
Solinviviridae
Marnaviridae
Noraviridae
Iflaviridae
Dicistroviridae
Polycipiviridae
